# An Acute-on-Chronic Presentation of Pneumatosis Cystoides Intestinalis: A Case Report

**DOI:** 10.7759/cureus.106406

**Published:** 2026-04-03

**Authors:** Michael D Cubello, James Giannone

**Affiliations:** 1 General Surgery, University of New England College of Osteopathic Medicine, Portland, USA; 2 General Surgery, St. Joseph's Health Hospital, Syracuse, USA

**Keywords:** computed tomography, exploratory laparotomy, pneumatosis cystoides intestinalis, pneumatosis intestinalis, pneumoperitoneum

## Abstract

Pneumatosis cystoides intestinalis (PCI) is a rare manifestation of pneumatosis intestinalis (PI), characterized by intramural gas cysts within the gastrointestinal tract. While frequently benign and many times idiopathic, PCI can also be associated with underlying critical conditions. Radiographically, benign cases of PCI can mimic life-threatening abdominal pathology, creating diagnostic uncertainty and challenging operative decision-making. We report a case of a 71-year-old male with a three-year history of progressive abdominal bloating, early satiety, postprandial pain and significant weight loss who presented acutely with worsening abdominal symptoms following a mechanical fall. Computed tomography (CT) revealed extensive small bowel pneumatosis with associated pneumoperitoneum. Despite concerning imaging findings, the patient lacked peritoneal signs, laboratory abnormalities, or evidence of mesenteric ischemia on computed tomography angiography (CTA). Given persistent symptoms and worsening radiologic appearance, an exploratory laparotomy was pursued. Intraoperative findings demonstrated a segment of dilated, atonic small bowel containing diffuse intramural air cysts without evidence of ischemia, perforation, obstruction, or purulent peritonitis. Small bowel resection with primary anastomosis was performed. The patient recovered uneventfully and experienced complete resolution of chronic gastrointestinal symptoms at postoperative follow-up. This case highlights the importance of integrating clinical presentation with laboratory and radiologic findings when evaluating pneumatosis intestinalis. Although small bowel involvement is often associated with pathologic etiologies, PCI may present as an acute exacerbation of chronic disease. Selective surgical intervention can be both diagnostic and therapeutic in symptomatic patients.

## Introduction

Pneumatosis cystoides intestinalis (PCI) is a condition defined by the presence of gas in the submucosal space in any portion of the gastrointestinal tract. Collections of gas bubbles may be identified radiographically or on gross pathology and represent an abnormal finding often associated with underlying disease. PCI is a subtype of pneumatosis intestinalis (PI), which is a broader term used to describe intramural free air within the intestines [1].

Most individuals with PCI are asymptomatic, making its true prevalence difficult to quantify; however, an incidence rate of 0.03% has been reported in the general population. PCI is most commonly seen between the fifth and eighth decades of life and demonstrates a male-to-female predominance of 3:1 [2]. Pneumatosis intestinalis can develop from bowel necrosis, various pulmonary conditions, abnormal mucosal permeability, organ transplantation, HIV, Crohn’s disease, chemotherapy, corticosteroids, trauma, systemic sclerosis among other autoimmune diseases, iatrogenic or idiopathic causes [1-4]. In pediatric populations, PCI is found to be more frequently associated with malignancy, autoimmune disorders and disorders of gastrointestinal motility, often in the setting of corticosteroid or immunosuppressive therapy [5].

PCI may be further classified based on both location and etiology. When pneumatosis is confined to the submucosa, PCI may be designated as the primary pathology. Additionally, PCI can be further categorized as primary or secondary. Primary PCI is characterized by benign, cystic intramural gas collections, whereas secondary PCI occurs in association with an underlying disease process, as described above [1]. Approximately 85% of PCI cases are secondary to another condition [2]. Clinical manifestations of primary PCI are often nonspecific and vary widely, with commonly reported symptoms including abdominal pain (59%), diarrhea (53%), nausea or vomiting (14%), mucus in stool (12%) and hematochezia (12%) [6]. Secondary PCI may present with similar gastrointestinal symptoms in addition to manifestations related to the underlying condition [1-4]. Serious complications occur in approximately 3% of PCI cases and include volvulus, intestinal obstruction, intestinal ischemia and pneumoperitoneum [6].

## Case presentation

A 71-year-old male with a past medical history of atrial fibrillation, on Eliquis, coronary artery bypass graft, Mobitz type 1 second-degree atrioventricular block, spinal stenosis, gastroesophageal reflux disease (GERD) and hypertension presented to St. Joseph’s Health Hospital in Syracuse, New York, one day after a mechanical fall to his right hip. He also had lower back pain, painful abdominal bloating, nausea and vomiting. Prior to this episode, the patient had a three-year history of abdominal bloating, decreased appetite, pain with meals and 70 pounds of unintentional weight loss. The patient’s bloating and abdominal pain significantly worsened subsequently after he fell, prompting his presentation to the emergency department.

The patient reported no previous history of abdominal surgery and followed up with his outpatient physicians regularly, who were unable to identify the origin of his complaints. He completed an esophagogastroduodenoscopy (EGD) and colonoscopy in 2021. Upon chart review, the EGD impression stated the patient had mucosa suggestive of Barrett’s esophagus, normal stomach, normal duodenum and normal jejunum. The colonoscopy impression stated normal mucosa in the terminal ileum, normal mucosa in the whole colon and external hemorrhoids. No intraoperative images were available for this procedure, as it was performed at an outside institution and the original procedural images were not accessible through our electronic medical record.

On initial evaluation, he appeared calm, in no apparent distress, with vital signs: 36.5°C, 53 heart rate (HR), 18 respiratory rate (RR) and 129/54 mmHg. Physical exam revealed abdominal distension and mild periumbilical tenderness without guarding or rebound tenderness. Lab work demonstrated a normal WBC, normal lactic acid, creatinine of 1.26, hemoglobin of 11.5 and potassium of 3.1. None of these values were highly concerning in relation to his acute abdominal pain. A full breakdown of the patient’s laboratory values at presentation can be found below in Table 1.

**Table 1:** Laboratory results: complete blood count, complete metabolic panel and lactate. Abnormal results are highlighted in bold.

Test	Result	Reference	Units
WBC	6	4.1-11.0	K/mcL
Hemoglobin	11.5	13.5-18	g/dL
Hematocrit	34.5	41.0-53.0	%
Platelets	137	150-450	K/mcL
Sodium	141	136-145	mmol/L
Potassium	3.1	3.5-5.1	mmol/L
Chloride	111	98-107	mmol/L
CO_2_	19	20-31	mmol/L
Blood urea nitrogen	23	9-23	mg/dL
Creatinine	1.26	0.70-1.30	mg/dL
Estimated glomerular filtration rate	61	≥60	mL/min/1.73 m^2^
Glucose	91	70-99	mg/dL
Aspartate transaminase (AST)	26	13-40	unit/L
Alanine transaminase (ALT)	23	9-40	unit/L
Alkaline phosphatase (ALP)	142	50-136	unit/L
Bilirubin, total	1.2	0.0-1.0	mg/dL
Calcium	6.8	8.3-10.6	mg/dL
Albumin	3.7	3.2-4.5	g/dL
Lactate	1.1	0.4-2.0	mmol/L

**Figure 1:**
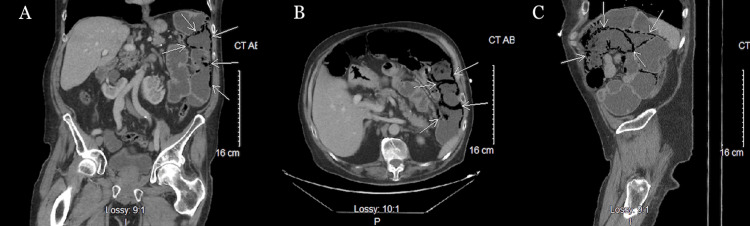
CT scan showing PCI in the left upper quadrant of the abdomen. A) Coronal view. B) Axial view. C) Sagittal view. This is an original CT scan of the patient detailed by this report. Pathology indicated by white arrows. PCI: pneumatosis cystoides intestinalis.

A computed tomography (CT) abdomen and pelvis with contrast was performed and displayed extensive pneumatosis intestinalis of the small intestine in the left upper quadrant and pneumoperitoneum (Figure 1).

**Figure 2:**
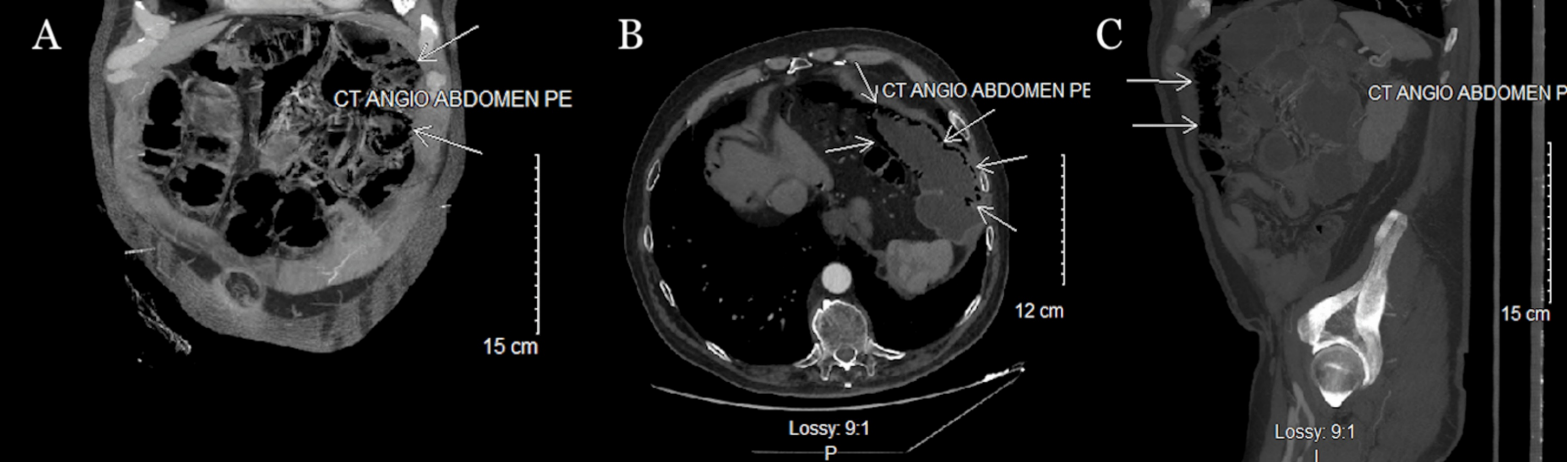
CTA scan showing increased bowel pneumatosis and dilation. A) Coronal view. B) Axial view. C) Sagittal view. This is an original CTA scan of the patient detailed by this report. Pathology indicated by white arrows. CTA: computed tomography angiography.

A diagnostic challenge arose due to discordance between the patient’s mostly benign physical examination, including the absence of peritonitis, and imaging findings demonstrating extensive pneumatosis and mild pneumoperitoneum, which was suspected to be due to scant cyst rupture. Prior to formulating an operative plan, a computed tomography angiography (CTA) of the abdomen and pelvis was obtained to evaluate for mesenteric ischemia, particularly given the patient’s history of atrial fibrillation. The CTA demonstrated increased pneumoperitoneum compared with the prior CT examination but revealed no evidence of vascular occlusion (Figure 2).

Due to the patient’s albeit mild abdominal pain and tenderness, but very concerning and worsening abdominal CT, exploratory laparotomy was discussed with the patient. Preoperative antibiotic prophylaxis was administered intravenously, including cefazolin 2 g and metronidazole 500 mg. During surgery, beginning at 20 cm distal to the ligament of Treitz, there was section of dilated, atonic, small bowel present that was continuous for a span of approximately 135 cm. There were no signs of perforation, no free fluid or contamination, no obstruction and no gross signs of ischemia; however, through this segment of small bowel, there were large air-filled sacs throughout the wall of the bowel. Without any other causes grossly apparent, healthy palpable mesenteric pulses present, and the clearly affected loop of small bowel, this portion was resected and a side-to-side stapled anastomosis was performed. No further exploration yielded any other etiology, and the surgery was completed. The patient tolerated the procedure well and was able to recover on the medical-surgical floor.

The pathology report was returned two days following the procedure. The final diagnosis detailed "a small bowel, segmental resection containing numerous mural cystic spaces lined by foreign body giant cells, consistent with pneumatosis cystoides intestinalis, acute enteritis, one benign lymph node." The comments further described acute inflammation of the intestinal walls, full thickness in some areas, and empty submucosal cysts lined by multinucleated giant cells which is consistent with air in the bowel. There were many cysts, spanning up to 0.5 cm in diameter at their largest size. The mesenteric adipose tissue was also dissected and showed unremarkable vasculature, consistent with the patency seen on the aforementioned CTA images.

The patient recovered uneventfully and was able to be discharged on post-op day six with resumption of bowel function, toleration of regular diet and no abdominal pain. At the two-week post-op appointment, the patient reported full resolution of all symptoms, stating that this was the best he has felt in years. His preoperative and chronic complaints of early satiety, appetite issues and pain after eating seem to have been resolved. At this time, he was given an abdominal binder to provide postoperative support, offered to follow-up as needed and released from surgical care. At four weeks post-op, this patient’s primary care physician (PCP) ordered a repeat set of labs to monitor his condition following surgery (Table 2).

**Table 2:** Laboratory results: complete blood count and complete metabolic panel. Abnormal results are highlighted in bold.

Test	Result	Reference	Units
WBC	4.9	4.1-11	K/mcL
Hemoglobin	9.1	13.5-18	g/dL
Hematocrit	28.4	41.0-53.0	%
Platelets	204	150-450	K/mcL
Sodium	145	136-145	mmol/L
Potassium	3.6	3.5-5.1	mmol/L
Chloride	108	98-107	mmol/L
CO_2_	22	20-31	mmol/L
Blood urea nitrogen	24	9-23	mg/dL
Creatinine	1.0	0.70-1.30	mg/dL
Estimated glomerular filtration rate	80	≥60	mL/min/1.73 m^2^
Glucose	117	70-99	mg/dL
Aspartate transaminase (AST)	15	13-40	unit/L
Alanine transaminase (ALT)	12	9-40	unit/L
Alkaline phosphatase (ALP)	102	50-136	unit/L
Bilirubin, total	1.0	0.0-1.0	mg/dL
Calcium	8.2	8.3-10.6	mg/dL
Albumin	3.5	3.2-4.5	g/dL

Despite some abnormal results, none were concerning at this time and at most mild in severity. His chronic health conditions were managed appropriately by his PCP during this visit. It is worth noting an improvement to the normal reference range in potassium, platelets, alkaline phosphatase and total bilirubin. A repeat order for lactate was unnecessary at this visit due to his significant clinical improvement. At six months post-op, a brief interview was held to obtain patient consent for this case report and to assess his recovery. The patient reported his condition remained stable, as he experienced no surgical complications or recurring symptoms. The patient did mention a weight gain of 30 pounds, which he attributed to his return to baseline in his appetite. He was satisfied with this result, and there was no need for further intervention or follow-up.

## Discussion

When evaluating acute abdominal pain in the emergency department, the initial goal is to assess for any life-threatening conditions. Among the potential etiologies of PCI, mesenteric ischemia is the most lethal, given that tissue hypoperfusion hastily progresses to bowel necrosis [3]. This highlights the importance of distinguishing a benign versus pathologic etiology in the acute period. The results of the CT angiography in this case effectively ruled out any occlusive form of ischemia. With a three-year history of abdominal pain, decreased appetite, substantial weight loss and elements of his past medical history, including significant cardiac surgery, non-occlusive mesenteric ischemia should be considered on the differential. Ischemia of any kind in the midgut would lead to weakness of the intestinal wall and subsequent small bowel dilation. This patient’s fall may have provoked an acute, small perforation in the affected portion of the small bowel, leading to pneumoperitoneum and mild resultant symptoms, which would be enough to classify his case as an acute-on-chronic presentation of PCI. However, It is also likely that this could have been an idiopathic case of PCI, as the patient experienced similar symptoms three years prior to the fall, with no identifiable underlying cause and lack of ischemia on imaging. Unfortunately, no clear etiology was able to be definitively uncovered by the investigation of this case.

The decision to operate on this patient can also be supported by a large retrospective cohort study known as the Pneumatosis Intestinalis Predictive Evaluation Study (PIPES trials). This study showed that radiologic evidence of PI in the ileum was likely due to pathologic cause, while evidence of PI in the large intestine was likely benign [7]. Radiologic evidence in our case was extensive in the proximal small bowel (duodenum and jejunum), blurring the lines further as to whether or not the disease in our case was related to some underlying pathologic cause. The lack of positive physical exam findings, pertinent irregular laboratory values or vascular occlusion on CTA seem to support an idiopathic etiology in this case acutely exacerbated by the patient's fall. This atypical distribution may explain the markedly different clinical presentation observed in this case compared to others in the literature, thereby limiting its treatment applicability to other PCI cases. Nevertheless, this unusual presentation contributes to the novelty of the case and justifies its formal documentation. The traumatic rupture of cysts in idiopathic PCI poses a plausible reiteration of the findings from the PIPES trial by demonstrating potentially pathologic PI predominantly residing in the small intestine rather than in the large intestine. These claims support deeming our case an example of PCI that is both idiopathic and pathologic.

Prevalence data summarized by a subsequent review article has shown that in 919 cases of PCI, 42% was localized to the ileum, 36% was localized to the colon and the remaining 22% extended between areas of both the small and large intestines [8]. This data is helpful to direct clinicians suspecting PCI toward the areas of highest likelihood of encountering this disease. Some factors that are predictors of poor prognosis include a pH < 7.3, bicarbonate level < 20 mmol/L, lactate level >2 mmol/L and amylase level >200 U/L. An elevated lactate value is the strongest predictor of pathological PCI and poor outcomes [6]. Although not all these labs were ordered in our case, the patient’s lactate was below this threshold at 1.1 and he had a positive surgical outcome, which was consistent with the results of the aforementioned study.

**Figure 3:**
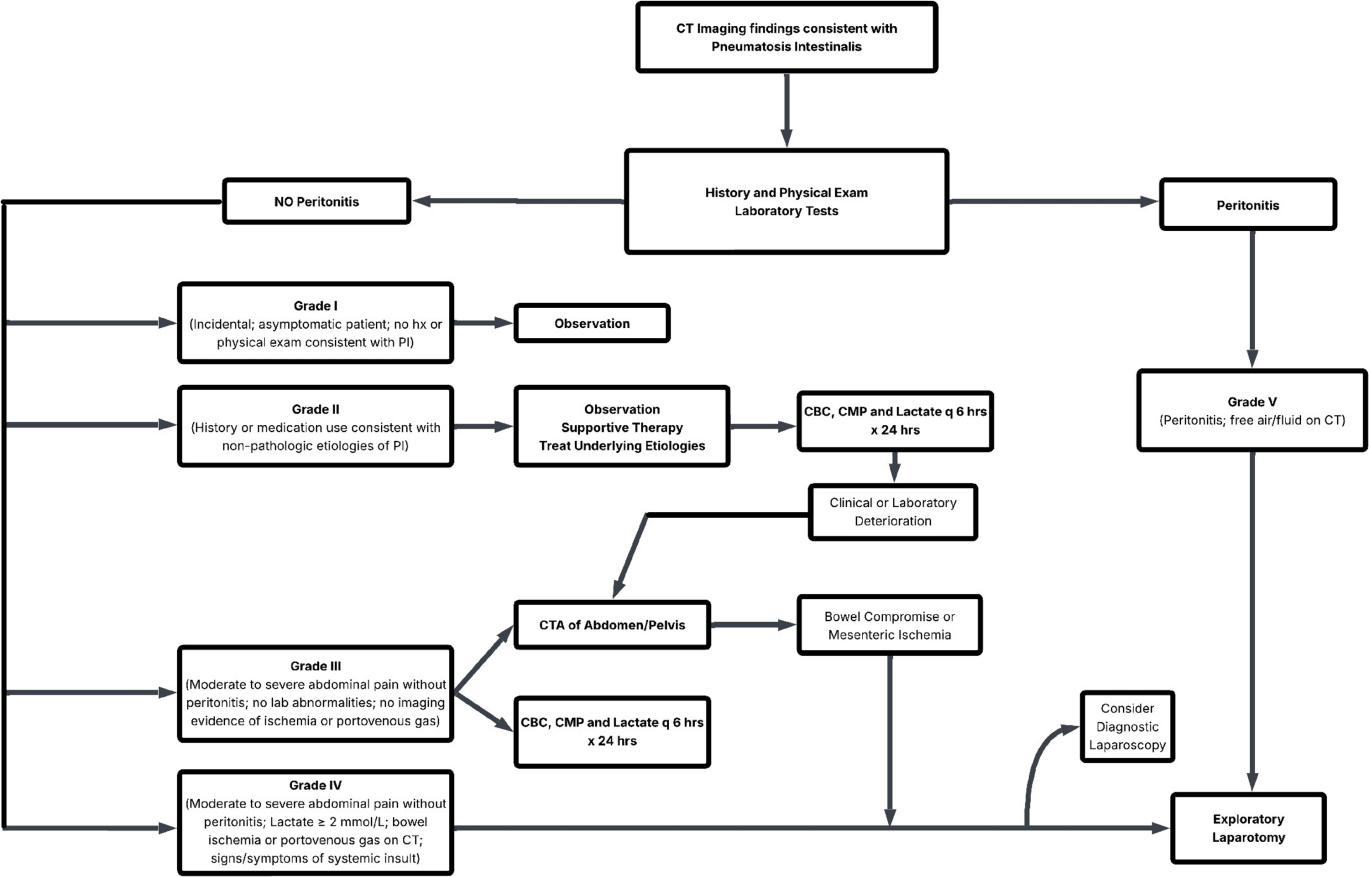
Flowchart for the management of pneumatosis intestinalis by grade. *Reprinted from Advances in Surgery, Vol 59, No. 1, Mather J, Diaz J, Pneumatosis intestinalis in adults, pp. 143-160, © 2025, with permission from Elsevier* [3]. hx: history, PI: pneumatosis intestinalis, CBC: complete blood count, CMP: complete metabolic panel, CTA: computed tomography angiography.

Our patient’s case was consistent with grade III pneumatosis intestinalis. This is defined as moderate to severe abdominal pain without peritonitis; no lab abnormalities; no imaging evidence of ischemia or portal venous gas [3]. The patient fit these criteria and thus followed the recommendation for a CTA abdomen and pelvis. This imaging, as previously mentioned, clearly showed bowel compromise, leading ultimately to exploratory laparotomy to resolve the condition. This process is outlined by the treatment algorithm referenced below (Figure 3).

Following exploratory laparotomy with small bowel resection, the patient’s condition improved clinically at two-week follow-up. This justifies the use of exploratory surgery as a mainstay of symptomatic management for this condition. In cases where bowel perfusion is of concern, injectable indocyanine green with imaging technology can adequately delineate perfused bowel from non-perfused bowel [3]. This patient had enough clinical and radiological findings to warrant exploratory surgery with small bowel resection to confirm PCI as a likely cause of his chronic complaints. Pneumoperitoneum is not always present in cases of PCI and its absence along with a lack of clinical peritonitis led to the use of diagnostic imaging studies.

Compared to pneumatosis intestinalis, PCI management is less definitive, and no standard treatment protocol has been implemented yet. Most cases of PCI have successfully been managed conservatively, with exploratory surgery reserved as a second-line therapy. Identifying the underlying cause of PCI is critical to treatment decision-making. A thorough analysis including a detailed history, physical exam, labs and radiological studies are necessary to rule out other underlying pathology [2].

In a radiology study, the use of CT with contrast (40 with IV contrast and two without contrast due to poor renal function) was used to compare asymptomatic (n=24) vs. symptomatic (n=18) patients with PI. The asymptomatic group was defined as having no symptoms or very mild abdominal symptoms, and the symptomatic group was defined as severe abdominal pain or unstable vital signs. Results showed that the symptomatic group had significantly increased bowel distension, decreased bowel wall enhancement, presence of portal venous gas and ascites. There was no difference between the groups for evidence of bowel wall defects, pneumoperitoneum or mesenteric venous gas [4]. Symptomatic patients with these additional symptoms were more at risk for life-threatening PI, whereas patients who were asymptomatic with radiologic evidence of PI were likely to be benign and unlikely to lead to any life-threatening condition. Out of the 18 patients in the life-threatening group, nine underwent immediate surgery due to bowel ischemia, one died shortly after completing their CT scan, four were recommended surgery but declined, three underwent antibiotic management and improved and one had a duodenal ulcer bleed that was endoscopically repaired successfully [4]. Therefore, those with red flags on CT paired with abdominal symptoms should strongly consider laparotomy; however, alternatives such as the use of antibiotics may be considered depending on the etiology of PI (e.g. gastroenteritis). Laparotomy is preferred over laparoscopy in this case to properly evaluate for extensive areas of PCI and enhance the removal of large segments of diseased bowel. Missing any compromised bowel would likely lead to recurrence of symptoms. This study helps guide treatment decision-making for surgeons based on radiological evidence and clinical examination of the patient. Our case did not rigidly meet the criteria for surgery proposed by this paper, but clinical judgement took precedence over the lack of red flag symptoms, such as portal venous gas, in our patient. This study highlights key details to evaluate for on CT imaging if PI is suspected but does not provide definitive management due to overlapping presentations of benign and life-threatening PI. In ambiguous cases such as this, the decision to operate ultimately is at the surgeon’s discretion.

There are conservative treatments described for PCI in patients who do not require urgent surgery; however, they may have chronic symptoms possibly attributable to other non-life-threatening etiologies such as asthma and chronic bronchitis. Secondary PCI due to pulmonary disease has been theorized to stem from alveolar rupture with air bubbles dissecting interstitially and traveling to the mediastinum and retroperitoneal space, eventually invading the perivascular spaces of intestinal walls. The use of metronidazole empirically has been reported as a viable non-surgical option in milder cases of PCI. Metronidazole is a highly effective and commonly used antibiotic with anaerobic coverage, which helps reduce intestinal bacterial hydrogen production [2]. In some cases, this may help to reduce the progression of PCI. The same article suggests use of hyperbaric oxygen therapy because it significantly elevates partial pressure of oxygen in arterial blood (PaO_2_). This induces a pressure gradient on the cyst gasses, forcing oxygen in, allowing the surrounding tissues to metabolize and resolve these cysts over time [2].

PCI however may be a truly idiopathic and incidentally discovered finding. If other more serious etiologies are ruled out due to lack of red flag signs (e.g. portal venous gas, arterial or venous occlusion, bowel wall thickening or altered contrast mucosal enhancement) and the patient is asymptomatic, then clinical observation may be an appropriate course. Imaging displaying PCI in this manner can safely be diagnosed as benign and likely idiopathic.

In a systematic review of 23 studies, 95 pediatric cases (age four months to 14 years) were compiled showing that PCI with pneumoperitoneum was detected mostly as an incidental finding on radiographic imaging in the setting of severe immunosuppression (e.g. malignancy, autoimmune disease, corticosteroid use). Of these 95 cases, 85% were managed conservatively (bowel rest, antibiotics, parenteral nutrition), 11% underwent exploratory laparotomy and only three were confirmed cases of bowel perforation [5]. This case demonstrated that surgery may not always be necessary for this condition. Classically, pneumoperitoneum has been a surgical emergency, but a majority of these cases were resolved conservatively. The authors of this review suggest that clinical stability, rather than imaging results alone, should guide the treatment approach [5].

While our report focuses on the diagnosis and treatment of PCI, the broader clinical applicability of this pathology is limited by several factors that reduce its generalizability to the general population. As previously noted, PCI is a rare subtype of PI; therefore, the majority of patients diagnosed with PI do not have PCI. In most cases, PCI is asymptomatic, requires no intervention and frequently remains undetected [2].

An additional limitation of this report is the absence of intraoperative photographic documentation of the gross pathology. Given the emergent nature of the operation and the lack of preoperative awareness of PCI in this patient, intraoperative images were not obtained. The novelty of the case was not recognized until postoperative review of the pathology report resulted. The lack of gross intraoperative imaging may hinder intraoperative recognition of similar pathology by other surgeons. Nevertheless, we were able to include representative CT images from our patient, which may aid future clinicians in recognizing and identifying PCI preoperatively, potentially improving diagnostic accuracy prior to deciding to undergo exploratory laparotomy.

## Conclusions

This case illustrates an appropriate diagnostic work-up and successful management of pneumatosis intestinalis, which ultimately was found to be an acute-on-chronic presentation of pneumatosis cystoides intestinalis (PCI). This can be a challenging diagnosis to determine, and without signs or symptoms of peritonitis, lab abnormalities, portal venous gas, ischemia on imaging or pneumoperitoneum, pneumatosis intestinalis has been reported to be managed non-operatively. Radiologic evidence is critical in diagnosing patients with PCI, as is a search for underlying causes. If any of these symptoms are identified, there should not be any delay in surgical intervention. Many cases of PCI are idiopathic in nature, but the goal is to rule out life-threatening diagnoses of pneumatosis intestinalis. Management with a stepped care approach relating to the patient presentation and clinically significant signs of destabilization can ensure patient safety with minimization of treatment complications.
